# Incremental predictive value of a CT-based deep learning radiomics model for differentiating benign and malignant pleural effusions

**DOI:** 10.3389/fmed.2026.1899111

**Published:** 2026-07-29

**Authors:** Chun Cao, Jiang Liu, Qingqing Fang, Tian Tian

**Affiliations:** 1 Department of Medical Imaging, Xinghua People’s Hospital Affiliated to Yangzhou University, Xinghua, China; 2 Department of Oncology, Xinghua People’s Hospital Affiliated to Yangzhou University, Xinghua, China

**Keywords:** biochemical biomarkers, deep learning, differential diagnosis, pleural effusion, radiomics

## Abstract

**Objective:**

To develop and validate a diagnostic model for malignant (MPE) and benign pleural effusion (BPE) using non-contrast chest CT deep learning (DL) and radiomics features, and to explore its incremental value alongside conventional biochemical biomarkers.

**Methods:**

We retrospectively enrolled 208 patients (Jan 2020–Sep 2024) as internal training/testing cohorts (7:3 ratio) and 52 patients (Oct 2024–Dec 2025) for internal temporal validation. Radiomics and DL features were extracted from non-contrast CTs to construct a radiomics score (Radscore). Multivariable logistic regression evaluated the Radscore’s independent predictive value. Net reclassification improvement (NRI) and integrated discrimination improvement (IDI) assessed the incremental diagnostic value when combined with clinical biomarkers.

**Results:**

Four radiomics features were ultimately retained to construct the Radscore. Multivariable adjusted analysis confirmed that the Radscore was an independent risk factor for MPE (OR: 2.718–2.776, *P* < 0.05). The model demonstrated good predictive performance (AUC = 0.805–0.864). Exploratory subgroup analyses indicated consistent discriminative trends, though estimates in certain strata were inherently limited by small event counts. Correlation analysis revealed only a weak correlation between pleural effusion carcinoembryonic antigen (pCEA) and the Radscore (*r* = 0.270, *P* = 0.001). Following the integration of pCEA, serum CEA (sCEA), pleural effusion adenosine deaminase (pADA), and serum CA125 (sCA125) into a combined clinical model, continuous NRI and IDI analyses demonstrated that the Radscore quantitatively refined individual risk estimation by shifting predicted probabilities in the appropriate direction for 75.0% of MPE patients and 66.7% of BPE patients.

**Conclusion:**

The DL-radiomics-based Radscore is a promising quantitative biomarker for differentiating MPE from BPE. It functions independently of conventional biochemical metrics and provides meaningful incremental value, refining the accuracy of risk probability estimation in patients with pleural effusion.

## 1 Introduction

Malignant Pleural Effusion (MPE) commonly arises from pleural metastasis of advanced malignancies such as Lung Cancer, Breast Cancer, and Lymphoma. Early and accurate differentiation between MPE and Benign Pleural Effusion (BPE) is clinically important because treatment planning and prognosis are closely related to the underlying etiology (1). At present, cytological examination of pleural effusion remains the standard approach for confirming MPE. However, the diagnostic sensitivity of cytology is limited, generally ranging from 40 to 70%, largely due to the heterogeneous distribution of malignant cells within the effusion or the presence of obscuring inflammatory components (2, 3). As a result, patients with suspected MPE but negative cytological findings often present a diagnostic dilemma in clinical practice. Conservative follow-up may delay treatment, whereas direct pleural biopsy introduces additional procedural risks and potential complications. Under these circumstances, the development of reliable and noninvasive diagnostic tools may help improve patient stratification and support more appropriate clinical decision-making.

Previous studies have shown that biochemical markers in blood and PE, including carcinoembryonic antigen (CEA), lactate dehydrogenase (LDH), and adenosine deaminase (ADA), can be incorporated into clinical prediction models and may provide diagnostic value in distinguishing MPE (4, 5). Nevertheless, these biomarkers mainly reflect local or systemic metabolic alterations associated with the tumor microenvironment and are limited in their ability to characterize the macroscopic spatial heterogeneity and morphological growth patterns of PEs. With chest CT now routinely performed in patients with PE, advances in deep learning (DL)-based radiomics have enabled the extraction of subtle imaging features that are difficult to recognize by visual assessment alone (6–9). Medical imaging and biochemical indicators describe lesions from different perspectives, namely macroscopic structural characteristics and microscopic metabolic changes. As a result, these two types of information may provide complementary value, which could be important for further exploring the additional clinical utility of chest CT in the evaluation of pleural diseases.

Accordingly, this study was designed to achieve four main objectives. First, a DL-Radiomics model based on the Radscore was developed to differentiate MPE from BPE, and its diagnostic performance was further evaluated across different patient subgroups. Second, the relationship between biochemical markers and the Radscore was investigated to explore the potential biological relevance underlying radiomic features. Third, we assessed whether incorporation of the Radscore into conventional biochemical marker-based models could provide additional diagnostic benefit for patients with indeterminate PE. The study was conducted in accordance with the Imaging Biomarker Standardization Initiative framework and complied with the TRIPOD+AI reporting guidelines (Supplementary Information 1 and 2).

## 2 Materials and methods

### 2.1 Patients and outcome definition

In this retrospective study, patients who underwent non-contrast chest CT examinations for PE at our institution between January 2020 and September 2024 were enrolled as the training and testing sets, while patients examined between October 2024 and December 2025 were included as the temporal external validation set. The inclusion criteria were as follows: (1) PE confirmed by chest CT examination; (2) completion of diagnostic thoracentesis or pleural biopsy; and (3) availability of complete imaging and clinical data. Patients were excluded if they met any of the following conditions: (1) uncertain PE etiology; (2) missing imaging data or poor image quality; or (3) incomplete clinical information.

MPE was defined according to the following criteria: (1) detection of malignant cells in pleural fluid cytology or diagnostic pleural biopsy specimens (10); (2) confirmation of a primary malignancy or clinical evidence of metastatic dissemination after exclusion of other possible causes of PE. This study was conducted in accordance with the Declaration of Helsinki and received approval from the institutional ethics committee (JSXHRYLL-NK-202463).

### 2.2 Clinical characteristics and imaging parameters

The following clinical and laboratory variables were collected for analysis: age, gender, smoking history, etiology of PE, leukocyte count, neutrophil count, monocyte count, lymphocyte count, platelet count, serum albumin level ( ≤ 55 vs. > 55 g/L), serum CEA (sCEA; ≤ 5 vs. > 5 ng/mL), serum LDH (sLDH; ≤ 250 vs. > 250 U/L), serum cancer antigen 125 (sCA125; ≤ 35 vs. > 35 U/mL), serum cancer antigen 19-9 (sCA199; ≤ 37 vs. > 37 U/mL), pleural effusion CEA (pCEA; ≤ 5 vs. > 5 ng/mL), pleural effusion LDH (pLDH; ≤ 274 vs. > 274 U/L), and pleural effusion ADA (pADA; ≤ 25.74 vs. > 25.74 U/L).

All patients underwent CT scanning using a 64-slice CT scanner (LightSpeed VCT, GE Healthcare). The scanning range extended from the thoracic inlet (upper margin of the T1 vertebral body) to the inferior margins of the bilateral adrenal glands. Scanning parameters were as follows: tube voltage, 120 kV; automatic tube current modulation; pitch, 0.8; collimation, 128 × 0.625 mm; slice thickness, 5 mm; and reconstruction interval, 5 mm. Based on the original data, thin-slice images were reconstructed with a slice thickness of 1.25 mm and an interval of 1.25 mm.

### 2.3 Workflow of deep learning and radiomics feature extraction

**FIGURE 1:**
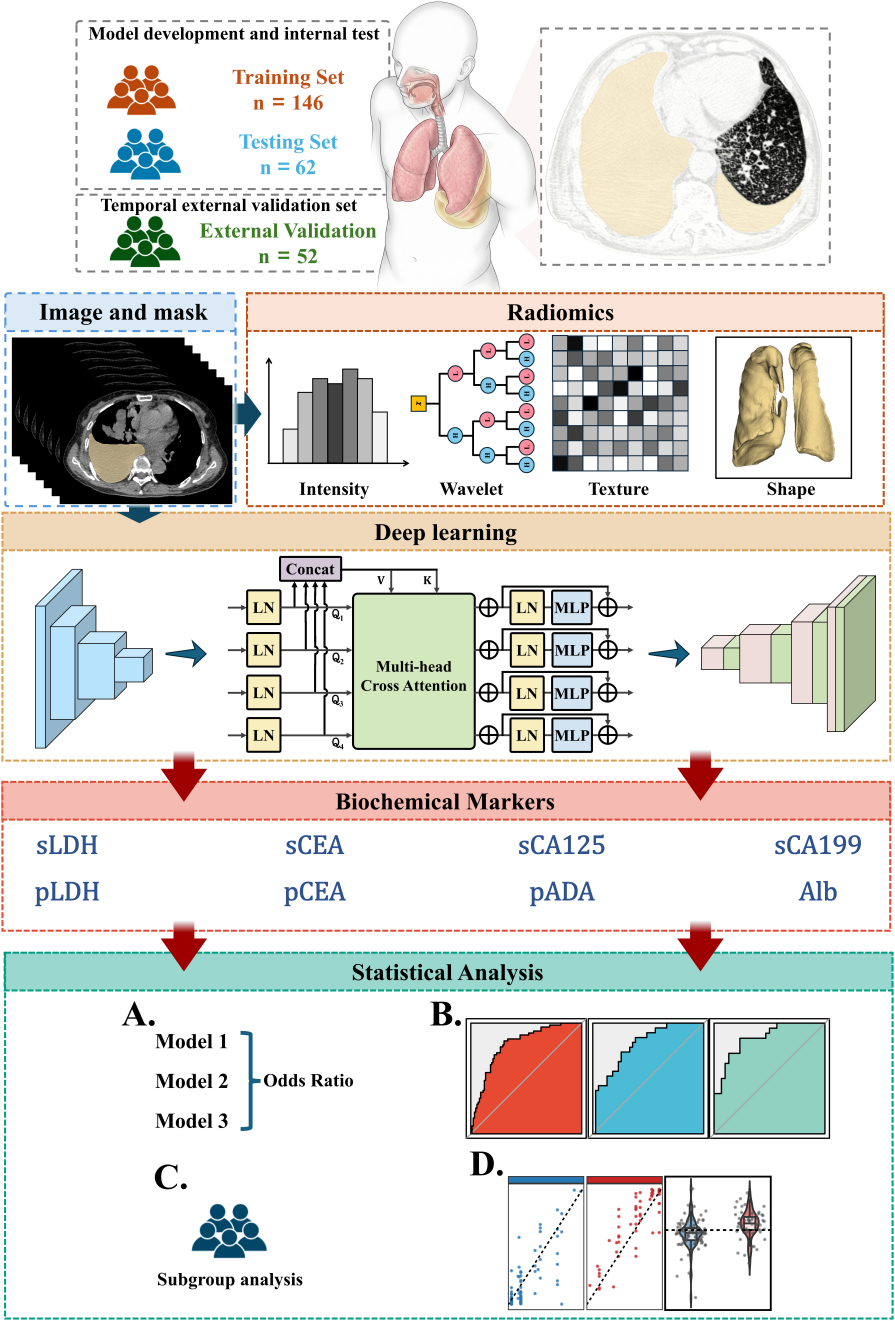
Flowchart of the CT-based deep learning radiomics model for incremental prediction of benign and malignant pleural effusions.

UCTransNet network was performed using a U-Net architecture integrated with a Channel Transformer module (11) (Figure 1). Each downsampling and upsampling block consisted of two grouped convolution layers. Every grouped convolution included a 3 × 3 convolution kernel, followed sequentially by batch normalization and a ReLU activation layer. Network parameters were optimized using the Adam optimizer with an initial learning rate of 0.0001 and a batch size of 32. The model showing the best validation performance was retained for subsequent feature extraction. A combined binary cross-entropy loss and Dice loss function was adopted during network training. The DL feature extraction workflow was conducted as follows. First, regions of interest (ROIs) were obtained for DL analysis, and all ROIs were resized to a standardized resolution of 224 × 224 pixels before being imported as input images. Second, min-max normalization was applied to the grayscale intensity values of the input images. Third, representative DL features were extracted from the normalized images by feeding them into the trained network. Feature maps were derived from the fourth downsampling activation layer of the U-Net model. Global average pooling was subsequently performed to generate a 1 × 512-dimensional semantic segmentation feature vector for each image. An unsupervised clustering algorithm was then applied to divide the extracted features into two clusters. The similarity between each cluster and the feature library was subsequently evaluated to identify the most informative feature combinations. In total, 512 DL features were extracted from each parametric map for every patient. The DL model was implemented using PyTorch 2.3.1 with CUDA 11.8 and was executed on an NVIDIA RTX 2080 Ti GPU (Supplementary Information 3).

Image preprocessing was performed prior to radiomics feature extraction. All images were resampled to isotropic voxels of 1 mm using B-spline interpolation, with a bin width of 5. ROI masks were resampled using nearest-neighbor interpolation. Because PE and pleural lesions may contain tissues with mixed attenuation values, an absolute threshold resegmentation strategy was applied, and voxel intensities were restricted to a CT attenuation range between −20 HU and 80 HU. Considering that Hounsfield units on CT images directly reflect the physical attenuation properties of tissues, global intensity normalization was not applied in order to preserve the original quantitative information and absolute reference scale of the images (normalization disabled; voxel array shift = 0). Radiomics features were extracted using PyRadiomics, including shape features, first-order statistics, gray-level co-occurrence matrix (GLCM), gray-level run-length matrix (GLRLM), gray-level size-zone matrix (GLSZM), gray-level dependence matrix (GLDM), and neighboring gray-tone difference matrix (NGTDM) features. Feature extraction was performed on original images as well as images processed using wavelet transformation and Laplacian of Gaussian (LoG) filters, resulting in a total of 851 radiomics features. The scaling parameters were calculated exclusively based on the training set. These exact, unchanged scaling parameters were subsequently applied to standardize the features in both the independent testing set and the temporal validation set prior to the application of the Radscore formula.

In this study, three-dimensional (3D) volumetric ROI segmentation was performed manually, slice-by-slice, across the entire axial extent of the effusion. All segmentations were conducted using the open-source software platform 3D Slicer (version 5.10.0). To ensure standardized anatomical boundaries, the ROI was strictly restricted to the fluid component of the pleural effusion. Adjacent anatomical structures and confounding pathologies were rigorously excluded. ROI delineation was initially performed by a radiologist with 6 years of clinical experience. To evaluate feature robustness and support subsequent feature selection, 30 patients were randomly selected from the training cohort and independently re-segmented by a senior radiologist with 13 years of experience. Inter-observer spatial overlap was quantified using the Dice Similarity Coefficient (DSC), and volumetric agreement was assessed using the Intraclass Correlation Coefficient (ICC).

### 2.4 Statistic analysis

Continuous variables were assessed for normal distribution using the Shapiro-Wilk test. Normally distributed continuous variables were expressed as mean ± standard deviation (SD) and compared across the three groups (training, testing, and validation cohorts) using analysis of variance (ANOVA). Skewed continuous variables were presented as median with interquartile range (IQR) and compared using the Kruskal-Wallis test. Categorical variables were presented as counts and percentages. Intergroup comparisons of categorical variables were evaluated using the Chi-square test; however, Fisher’s exact test was employed when the expected frequency in any cell was less than 5 due to sparse data. For all *post-hoc* pairwise comparisons across the three cohorts, the Bonferroni correction was rigorously applied to adjust the *P*-values and control the family-wise error rate. Dataset partitioning was performed using the createDataPartition package. Sample size estimation was based on the 10 events per variable (10 EPV) principle (12).

To ensure reproducibility, features extracted from ROIs independently delineated by two radiologists were initially assessed using the ICC. Only features with an ICC > 0.75 and non-zero variance were retained. To strictly prevent data leakage, the subsequent supervised feature selection and performance evaluation were encapsulated within a 10-fold nested cross-validation (CV) framework on the training set. Within each outer iteration of the nested CV, the feature selection pipeline was exclusively fitted on the training folds and subsequently applied to the hold-out validation fold. This nested pipeline sequentially consisted of: (1) an F-test (*P* < 0.05) to preserve statistically significant features; (2) a correlation filter to remove highly redundant features (| r| > 0.9); and (3) a hybrid dimensionality reduction combining Least Absolute Shrinkage and Selection Operator (LASSO) regression and Support Vector Machine-Recursive Feature Elimination (SVM-RFE) with an inner CV loop. Finally, an unbiased estimate of the internal AUC was obtained. Following the unbiased performance estimation via nested CV, the final definitive Radscore model for external evaluation was established by applying this exact same multi-step feature selection pipeline (F-test, correlation filtering, LASSO, and SVM-RFE) to the entire training cohort globally. The ultimately selected features from this global pipeline were weighted by their respective Logistic Regression coefficients to construct the final Radscore (Figure 1 and Supplementary Figure 1).

Both unadjusted and adjusted logistic regression analyses were performed in the training cohort to evaluate the association between the Radscore and MPE. Adjustment was conducted sequentially using four models: Model 1 included the Radscore alone; Model 2 additionally adjusted for age and sex; Model 3 further incorporated sCEA, sCA125, pCEA, and pADA. The predictive performance of the Radscore was evaluated using the area under the receiver operating characteristic curve (AUC) and confusion matrix analysis. Calibration curves and decision curve analysis (DCA) were further generated to evaluate model calibration and potential clinical utility. Subgroup analyses were conducted to investigate the performance of the Radscore under different clinical conditions. Associations between the Radscore and PE biochemical markers were also explored. In addition, clinical predictors for MPE were identified using LASSO regression and multivariable logistic regression to construct a clinical prediction model. Net reclassification improvement (NRI) and integrated discrimination improvement (IDI) analyses were subsequently performed to evaluate the incremental diagnostic value provided by the incorporation of the Radscore. A two-sided *P* value < 0.05 was considered statistically significant. All statistical analyses were performed using RStudio software (v5.1.2) and Python (v3.12).

## 3 Results

### 3.1 Baseline characteristic

A total of 260 patients with PE were included in this study. Among them, 208 patients were assigned to the internal cohort, including the training set (*n* = 146) and testing set (*n* = 62), while 52 patients were enrolled in the temporal validation cohort (Supplementary Figure 2). MPE was identified in 101 patients (38.85%). The EPV calculation was based strictly on the number of minority outcome events (MPE) in the training cohort (7:3 split). In the training set (*n* = 146), 56 MPE events were observed; therefore, the final model was restricted to no more than five features. No significant differences were observed in baseline characteristics among the training, testing, and temporal validation cohorts (*P* > 0.05). Detailed comparisons of patient characteristics across the three cohorts are presented in Table 1, while comparisons between the BPE and MPE groups are provided in Supplementary Table 1.

**TABLE 1:** Comparison of characteristics among the training, testing, and validation sets.

Variables	Training set (*n* = 146)	Testing set (*n* = 62)	Validation set (*n* = 52)	*P* *
MPE, *n* (%)				0.837
No	90 (61.64)	39 (62.90)	30 (57.69)	
Yes	56 (38.36)	23 (37.10)	22 (42.31)	
Age (years)	70.32 ± 10.78	72.50 ± 10.78	72.44 ± 8.98	0.506
Gender (%)				0.875
Female	58 (39.73)	27 (43.55)	21 (40.38)	
Male	88 (60.27)	35 (56.45)	31 (59.62)	
Smoking (%)				0.498
No	125 (85.62)	49 (79.03)	43 (82.69)	
Yes	21 (14.38)	13 (20.97)	9 (17.31)	
Etiology (%)				0.358
Poorly differentiated	0 (0.00)	0 (0.00)	1 (1.92)	
Squamous carcinoma	2 (1.37)	1 (1.61)	0 (0.00)	
Adenocarcinoma	50 (34.25)	20 (32.26)	19 (36.54)	
Small cell carcinoma	1 (0.68)	0 (0.00)	1 (1.92)	
Breast cancer	3 (2.05)	1 (1.61)	1 (1.92)	
Lymphoma	0 (0.00)	1 (1.61)	0 (0.00)	
Tuberculosis	19 (13.01)	12 (19.35)	10 (19.23)	
Parapneumoniceffusion	43 (29.45)	19 (30.65)	14 (26.92)	
Heart failure	4 (2.74)	4 (6.45)	3 (5.77)	
Cirrhosis	15 (10.27)	4 (6.45)	3 (5.77)	
Other benign diseases	9 (6.16)	0 (0.00)	0 (0.00)	
Leukocyte (10^9^/L)	6.25 (5.13, 7.64)	6.42 (5.18, 8.63)	6.35 (5.30, 7.30)	0.668
Neutrophil (10^9^/L)	4.95 (3.90, 7.58)	4.90 (3.83, 6.78)	5.35 (4.20, 7.35)	0.755
Monocyte (10^9^/L)	0.50 (0.34, 0.66)	0.48 (0.36, 0.64)	0.46 (0.34, 0.57)	0.412
Lymphocyte (10^9^/L)	0.90 (0.60, 1.20)	0.90 (0.70, 1.38)	0.90 (0.60, 1.13)	0.731
Platelet (10^9^/L)	168.00 (132.20, 240.80)	190.00 (133.20, 198.60)	188.0 (136.50, 249.50)	0.496
Albumin (g/L)				0.143
≤ 55	140 (95.89)	62 (100.00)	52 (100.00)	
> 55	6 (4.11)	0 (0.00)	0 (0.00)	
sLDH (U/L)				0.561
≤ 250	96 (65.75)	45 (72.58)	37 (71.15)	
> 250	50 (34.25)	17 (27.42)	15 (28.85)	
sCEA (ng/mL)				0.281
≤ 5	107 (73.29)	43 (69.35)	32 (61.54)	
> 5	39 (26.71)	19 (30.65)	20 (38.46)	
sCA125 (U/mL)				0.412
≤ 35	95 (65.07)	35 (56.45)	30 (57.69)	
> 35	51 (34.93)	27 (43.55)	22 (42.31)	
sCA199 (ng/mL)				0.939
≤ 37	126 (86.30)	55 (88.71)	45 (86.54)	
> 37	20 (13.70)	7 (11.29)	7 (13.46)	
pCEA (ng/mL)				0.581
≤ 5	104 (71.23)	43 (69.35)	33 (63.46)	
> 5	42 (28.77)	19 (30.65)	19 (36.54)	
pLDH (U/L)				0.102
≤ 274	57 (39.04)	24 (38.71)	12 (23.08)	
> 274	89 (60.96)	38 (61.29)	40 (76.92)	
pADA (U/L)				0.526
≤ 25.74	98 (67.12)	38 (61.29)	37 (71.15)	
> 25.74	48 (32.88)	24 (38.71)	15 (28.85)	
Radscore	−0.57 (−1.64, 0.42)	−0.34 (−1.37, 0.49)	−0.62 (−1.42, 0.41)	0.685

MPE, malignant pleural effusion; sLDH, serum lactate dehydrogenase; sCEA, serum carcinoembryonic antigen; sCA125, serum cancer antigen 125; sCA199, serum cancer antigen 199; pCEA, pleural effusion carcinoembryonic antigen; pLDH, pleural effusion lactate dehydrogenase; pADA, pleural effusion adenosine deaminase.

*Bonferroni correction.

### 3.2 Selection results of DL radiomics and clinical features

**FIGURE 2:**
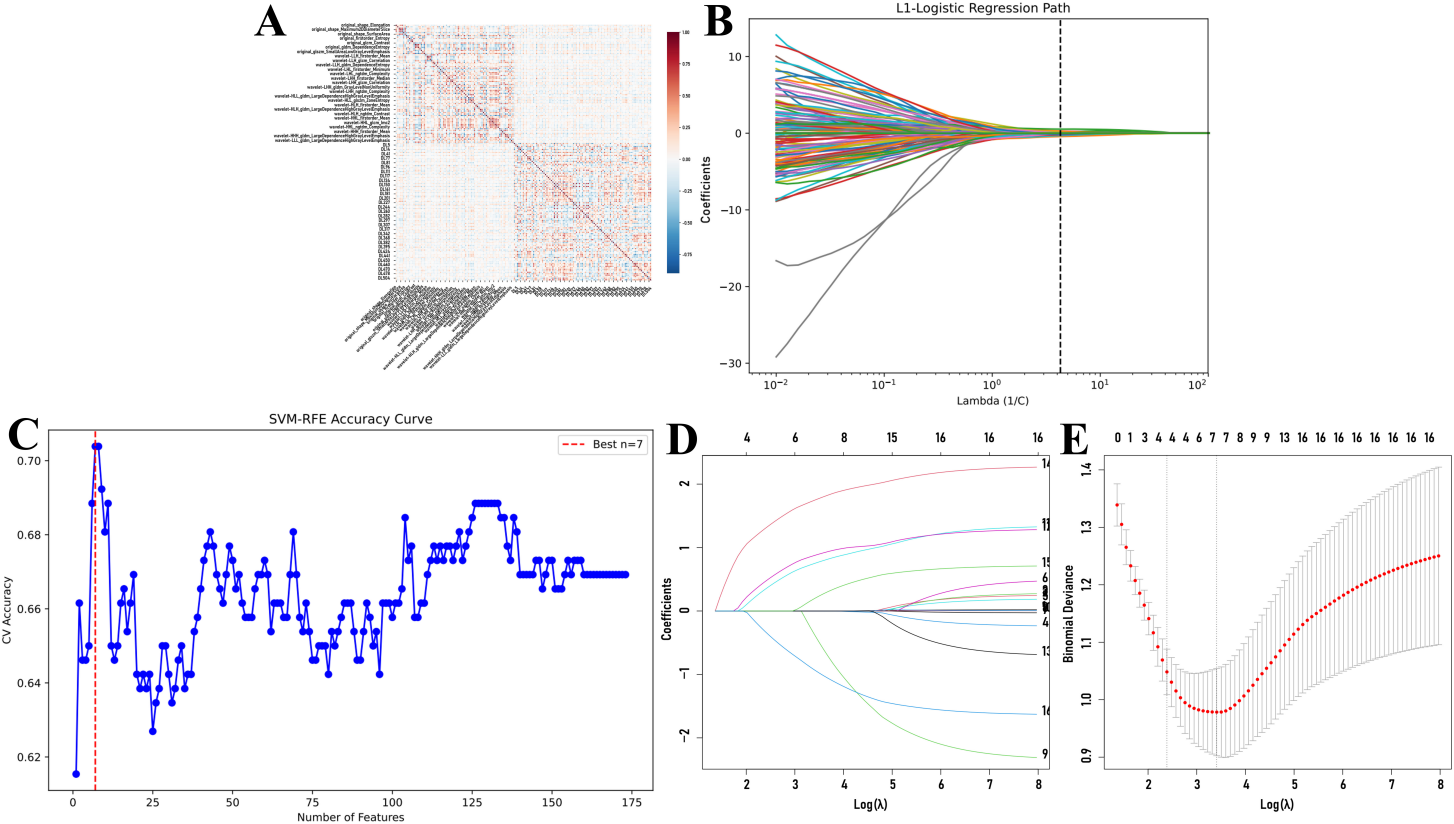
Results of LASSO regression and support vector machine recursive feature elimination (SVM-RFE) analyses for deep learning radiomics feature selection **(A–C)**, and LASSO regression analysis for clinical feature selection **(D,E)** . **(A)** Correlation matrix heatmap of deep learning radiomics features; **(B)** Variation in LASSO regression coefficients of deep learning radiomics features; **(C)** SVM-RFE accuracy and feature number plot for deep learning radiomics features; **(D,E)** Variation in LASSO regression coefficients of clinical features.

Following the feature selection process, four DL-Radiomics features were retained for model construction (Figure 2). The final Radscore was calculated according to the following formula:


$$\text{Radscore}=\beta_{0}+\beta_{1}\,{\text{F}}_{1}+\beta_{2}\,{\text{F}}_{2}+\beta_{3}\,{\text{F}}_{3}+\beta_{4}\,{\text{F}}_{4}$$

In the final model, β_0_ represented the intercept (-0.7112). F1 corresponded to DL_295 (DL imaging feature index No. 295) with a coefficient (β_1_) of 0.7734. F2 represented DL_4 (DL imaging feature index No. 4) with a coefficient (β_2_) of -0.6535. F3 corresponded to DL_80 (DL imaging feature index No. 80) with a coefficient (β_3_) of 0.7336. F4 represented wavelet-LLH_glcm_Idmn, namely the inverse difference moment normalized feature derived from the gray-level co-occurrence matrix under wavelet-LLH filtering, with a coefficient (β_4_) of 0.8488. The Dice similarity coefficient was 0.883, indicating good spatial consistency. In addition, absolute agreement of physical ROI volumes was assessed, yielding an ICC of 0.869 (95% CI: 0.710–0.940, Supplementary Figure 3).

According to the results of LASSO and multivariable logistic regression analyses (Figure 2), four clinical variables, including pCEA, sCEA, pADA, and sCA125, were ultimately selected for model construction.

### 3.3 Evaluation of the DL radiomics model

**FIGURE 3:**
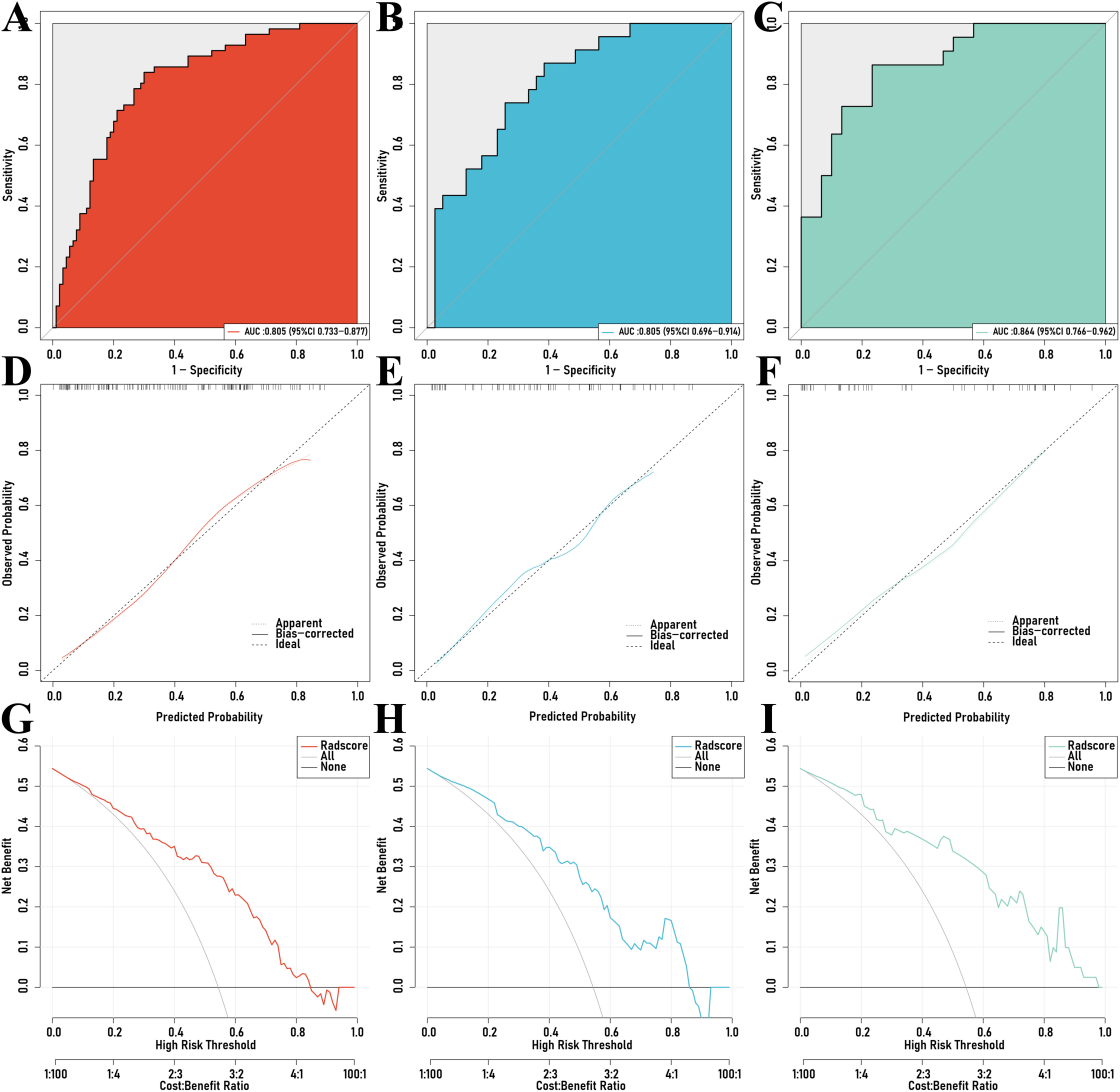
Receiver operating characteristic (ROC) curves **(A–C)**, calibration curves **(D–F)**, and decision curve analysis (DCA) curves **(G–I)** of the Radscore in the training, testing, and validation sets.

As shown in Table 2, the odds ratios (ORs) of the Radscore in both unadjusted and adjusted models ranged from 2.718 to 2.776 (95% CI 1.735 to 4.866). In the training set, the Radscore achieved an AUC of 0.805 (95% CI: 0.733–0.877), with an accuracy of 0.753, sensitivity of 0.839 (95% CI: 0.717–0.924), and specificity of 0.700 (95% CI: 0.594–0.792). In the testing set, the AUC was 0.805 (95% CI: 0.696–0.914), with an accuracy of 0.710, sensitivity of 0.870 (95% CI: 0.664–0.972), and specificity of 0.615 (95% CI: 0.446–0.776). In the temporal validation set, the model demonstrated an AUC of 0.864 (95% CI: 0.766–0.962), with an accuracy of 0.808, sensitivity of 0.864 (95% CI: 0.651–0.971), and specificity of 0.767 (95% CI: 0.577–0.901) (Figure 3 and Table 3). The unbiased internal mean AUC expectedly adjusted to 0.758 (Supplementary Figure 1). Calibration curves for the Radscore were generated using 1,000 bootstrap resampling iterations (Figure 3). The calibration analysis demonstrated good agreement between predicted probabilities and observed outcomes. DCA demonstrated that the model provided additional net clinical benefit across threshold probabilities of 0.07–0.86 in the training cohort, 0.05–0.87 in the testing cohort, and 0.02–0.98 in the validation cohort (Figure 3). During internal validation, the Radscore maintained stable predictive performance.

**TABLE 2:** Logistic regression analysis of Radscore across different models.

Model	*B*	*S.E*	*P*	*OR*	95% CI	
					Lower	Upper
Model 1	1.000	0.189	< 0.001	2.718	1.928	4.057
Model 2	1.013	0.191	< 0.001	2.755	1.947	4.129
Model 3	1.021	0.261	< 0.001	2.776	1.735	4.866

Model 1: Radscore; Model 2: Model 1, Age, Gender; Model 3: Model 2, sCEA, sCA125, pCEA, pADA.

**TABLE 3:** Diagnostic performance of the Radscore in the training, testing, and internal validation cohorts.

Dataset	Sample size	AUC (95% CI)	Accuracy	Sensitivity (95%CI)	Specificity (95%CI)
Training set	146	0.805 (0.733, 0.877)	0.753	0.839 (0.717, 0.924)	0.700 (0.594, 0.792)
Testing set	62	0.805 (0.696, 0.914)	0.710	0.870 (0.664, 0.972)	0.615 (0.446, 0.766)
Validation set	52	0.864 (0.766, 0.962)	0.808	0.864 (0.651, 0.971)	0.767 (0.577, 0.901)

### 3.4 Subgroup analysis and clinical value of the DL radiomics model

Subgroup analyses are summarized in Table 4. The Radscore maintained general discriminative ability across demographic subgroups, achieving AUCs of 0.827 (95% CI: 0.694–0.960; 12 MPE events) and 0.817 (95% CI: 0.761–0.872; 89 MPE events) in the ≤ 60 and > 60 age groups, respectively. In female and male patients, the AUCs were 0.833 and 0.803. When stratified by clinical biomarkers, the AUCs ranged from 0.733 (pCEA > 5 ng/mL) to 0.853 (pCEA ≤ 5 ng/mL). None of the interaction *P*-values indicated a statistically significant difference between the strata (Age: *P* = 0.890; Gender: *P* = 0.577; pCEA: *P* = 0.124; pLDH: *P* = 0.290; pADA: *P* = 0.619).

**TABLE 4:** Subgroup analysis of Radscore.

Subgroup	Sample size	MPE Events	AUC (95%CI)	Accuracy	Sensitivity (95%CI)	Specificity (95%CI)	PPV (95%CI)	NPV (95%CI)	*P*_*Interaction*_
Age (years)									0.890
≤ 60	39	12	0.827 (0.694, 0.960)	0.821	0.750 (0.428, 0.945)	0.852 (0.663, 0.958)	0.692 (0.386, 0.909)	0.885 (0.698, 0.976)	
> 60	221	89	0.817 (0.761, 0.872)	0.756	0.843 (0.750, 0.911)	0.697 (0.611, 0.774)	0.652 (0.558, 0.739)	0.868 (0.788, 0.926)	
Gender (%)									0.577
Female	106	43	0.833 (0.752, 0.914)	0.793	0.791 (0.640, 0.900)	0.794 (0.673, 0.885)	0.723 (0.574, 0.844)	0.847 (0.730, 0.928)	
Male	154	58	0.803 (0.735, 0.871)	0.760	0.810 (0.686, 0.901)	0.729 (0.629, 0.815)	0.644 (0.523, 0.753)	0.864 (0.770, 0.930)	
pCEA (ng/mL)									0.124
≤ 5	180	41	0.853 (0.794, 0.913)	0.750	0.927 (0.801, 0.985)	0.698 (0.614, 0.773)	0.475 (0.362, 0.590)	0.970 (0.915, 0.994)	
> 5	80	60	0.733 (0.592, 0.873)	0.750	0.800 (0.677, 0.892)	0.600 (0.361, 0.809)	0.857 (0.738, 0.936)	0.500 (0.291, 0.709)	
pLDH (U/L)									0.290
≤ 274	93	29	0.767 (0.666, 0.867)	0.699	0.724 (0.528, 0.873)	0.688 (0.559, 0.798)	0.512 (0.351, 0.671)	0.846 (0.719, 0.931)	
> 274	167	72	0.831 (0.769, 0.892)	0.778	0.903 (0.810, 0.960)	0.684 (0.581, 0.776)	0.684 (0.581, 0.776)	0.903 (0.810, 0.960)	
pADA (U/L)									0.619
≤ 25.74	173	91	0.824 (0.762, 0.886)	0.780	0.857 (0.768, 0.922)	0.695 (0.584, 0.792)	0.757 (0.663, 0.836)	0.814 (0.703, 0.897)	
> 25.74	87	10	0.783 (0.637, 0.930)	0.782	0.800 (0.444, 0.975)	0.779 (0.670, 0.866)	0.320 (0.149, 0.535)	0.968 (0.888, 0.996)	

pCEA, pleural effusion carcinoembryonic antigen; pLDH, pleural effusion lactate dehydrogenase; pADA, pleural effusion adenosine deaminase.

**FIGURE 4:**
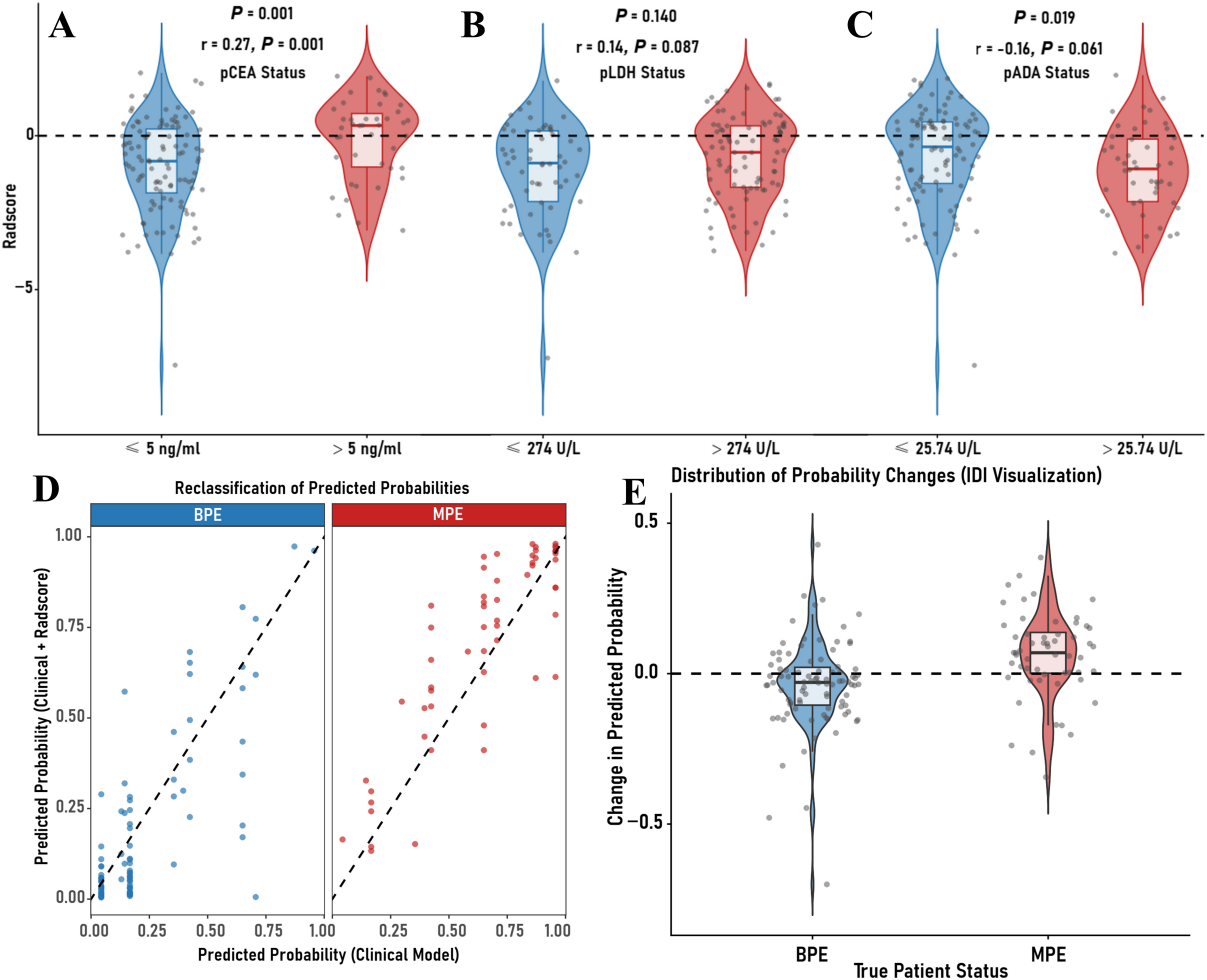
Correlation analysis between biochemical markers of pleural effusion and the Radscore **(A–C)**, as well as the net reclassification index **(D)** and integrated discrimination improvement analysis **(E)** comparing the combined model (clinical model + Radscore) with the clinical model alone.

The Radscore showed a positive correlation with pCEA levels (*r* = 0.270, *P* = 0.001; Figure 4). Although significantly higher Radscore values were observed in patients with elevated pLDH levels and reduced pADA levels (both *P* < 0.05), no significant linear correlation was identified between these variables (*P* > 0.05; Figure 4). In addition, the Radscore demonstrated superior diagnostic performance compared with other biochemical markers, as reflected by its higher AUC values (Supplementary Figure 4). After incorporation of the Radscore into the clinical model, a significant improvement in risk stratification was observed. The overall continuous NRI was 0.833 (95% CI: 0.534–1.132, *P* < 0.001; Figure 4 and Table 5). Specifically, the combined model shifted the predicted probabilities in the correct direction for 75.0% of patients with MPE (event NRI = 0.500, *P* < 0.001) and decreased the predicted probabilities for 66.7% of patients with BPE (non-event NRI = 0.333, *P* < 0.001). It is important to note that as a category-free metric, this reflects directional shifts in estimated probabilities rather than reclassification across predefined clinical thresholds. Similarly, IDI analysis demonstrated that the mean difference in predicted probabilities between MPE and BPE patients increased by approximately 9.90% after the inclusion of the Radscore (95% CI: 0.050–0.147, *P* < 0.001; Table 5 and Figure 4). The overall NRI in the temporal validation set was 0.588 (*P* = 0.025), and the IDI was 0.183 (*P* < 0.001, Supplementary Table 2). The combined model achieved AUCs of 0.922 (95% CI: 0.880–0.965), 0.929 (95% CI: 0.867–0.990), and 0.944 (95% CI: 0.877–1.000) in the training, testing, and validation sets, respectively (Supplementary Table 3).

**TABLE 5:** Reclassification and discrimination improvement of the combined model (clinical model + Radscore) compared with the clinical model alone.

Metrics	Estimate	SE	Z	95% CI	*P*
Continuous NRI					
Overall NRI	0.833	0.153	5.46	0.534, 1.132	< 0.001
NRI for events	0.500	0.116	4.32	0.273, 0.727	< 0.001
Increase for events	75.0%	–	–	–	–
Decrease for events	25.0%	–	–	–	–
NRI for non-events	0.333	0.099	3.35	0.139, 0.528	< 0.001
Decrease for non-events	66.7%	–	–	–	–
Increase for non-events	33.3%	–	–	–	–
IDI					
Overall IDI	0.099	0.025	3.97	0.050, 0.147	< 0.001-
Mean change in events	0.0609	–	–	–	–
Mean change in non-events	0.0379	–	–	–	–

NRI, net reclassification index; IDI, integrated discrimination improvement.

Formal pairwise comparisons of model discrimination (Supplementary Table 4) demonstrated that the Combined Model significantly outperformed the Clinical Model in both the training and testing sets (both *P* < 0.05, DeLong’s test). In the validation cohort, the combined model showed a *P*_*Delong*_ value of 0.053 compared with the clinical model. Quantitative calibration metrics (Supplementary Table 5) further demonstrated superior calibration performance of the combined model, with a lower Brier score (0.112 vs. 0.157) and a calibration slope closer to the value of 1 (0.977 vs. 0.901) than the clinical model. Furthermore, the Radscore alone did not show statistical superiority over the Clinical Model across the cohorts (all *P* > 0.05).

## 4 Discussion

Accurate early differentiation between MPE and BPE remains a major challenge in clinical decision-making. In the present study, the Radscore derived from DL and radiomics features was identified not only as a stable independent risk factor for MPE, but also as a valuable complement to conventional biochemical prediction models. More importantly, incorporation of the Radscore substantially improved the overall diagnostic performance of the traditional clinical model. Our findings demonstrated only weak or non-significant correlations between the Radscore and commonly used biochemical markers, including pCEA, pLDH, and pADA. This observation suggests that radiomics features may capture lesion heterogeneity that is not reflected by conventional metabolic or biochemical indicators. Owing to this relatively low degree of information overlap, the addition of the Radscore provided meaningful incremental value for risk stratification. In particular, when evaluated in the independent temporal validation cohort, the combined model achieved favorable continuous NRI and IDI, especially among patients with negative cytology results. It is crucial to emphasize that rather than strictly reclassifying a significant proportion of patients across predefined clinical decision thresholds, these continuous metrics specifically indicate that the integration of the Radscore quantitatively refined individual risk estimations by shifting predicted probabilities in a favorable direction (i.e., assigning higher probabilities to true MPE events and lower probabilities to non-events). Furthermore, the model demonstrated relatively stable predictive performance across multiple clinical subgroups within the temporal validation cohort. However, because the number of MPE events was limited in several specific subgroups, these subgroup analyses should be interpreted strictly as exploratory evidence.

Pleural fluid cytology remains the preferred diagnostic approach for confirming MPE. However, the distribution of malignant cells within pleural fluid samples is often heterogeneous, and tumor cells may occasionally be obscured by inflammatory or background cellular components, which can reduce diagnostic sensitivity. As a result, increasing attention has been directed toward identifying additional indicators from PE analysis to improve diagnostic accuracy, and several previous studies have reported good results (13, 14). For example, Wang SF et al. (4) developed a predictive model incorporating fever, erythrocyte sedimentation rate, pADA, sCEA, and pCEA, which demonstrated excellent diagnostic performance, with an internal temporal validation AUC of 0.912. Similarly, Wang SY et al. (5) developed a model based on neuron-specific enolase (NSE), cytokeratin 19 fragment, pCEA, and sCEA, achieving an AUC of 0.936 in the validation cohort. With the rapid development of radiomics, increasing attention has been paid to the clinical value of opportunistic information embedded within routinely acquired medical imaging data (15). Radiomics analysis based on PE-related imaging has also shown good results in recent years (16, 17). For instance, Cai et al. (8) developed a Radscore using 15 radiomics features derived from PE images, achieving an AUC of 0.820 in the testing cohort. In the same study, a clinical prediction model incorporating age, laterality of effusion, procalcitonin, sCEA, sCA125, and NSE also achieved an AUC of 0.820, while the combined model further improved the AUC to 0.870. Similarly, Han et al. (9) developed a Radscore based on 19 radiomics features, which yielded an AUC of 0.808 in the testing cohort. Their biomarker-based clinical model, constructed using sCEA, pCEA, serum lactate dehydrogenase, and PE adenosine deaminase, achieved an AUC of 0.764, whereas the integrated model reached an AUC of 0.873. In the present study, the Radscore derived from three DL features and one radiomics feature achieved an AUC of 0.864 in the temporal validation cohort. More importantly, incorporation of the Radscore effectively reduced risk misclassification generated by the clinical model based on pCEA, sCEA, pADA, and sCA125. This may help decrease unnecessary invasive procedures in patients with true BPE, while also reducing missed diagnoses and treatment delays in patients with MPE. These findings are consistent with our hypothesis that radiomics can provide heterogeneous lesion information that is relatively independent of the microscopic metabolic characteristics reflected by conventional biochemical markers. Notably, no additional examinations were required in this study, as all imaging information was obtained from routine non-contrast chest CT scans already performed in clinical practice.

To further explore the potential biological relevance of the Radscore, correlation analyses were performed between the Radscore and biochemical markers in PE. We observed significant distribution differences in the Radscore across pCEA and pADA subgroups, while only a weak positive correlation was identified between the Radscore and pCEA. Marked elevation of pADA is commonly considered a characteristic finding in tuberculous pleuritis (18). Accordingly, the low-pADA subgroup in this study largely consisted of patients with MPE. Because the Radscore itself tended to be higher in MPE cases, higher Radscore values were also observed in the low-pADA subgroup, which was consistent with the underlying disease distribution. Similarly, elevated pCEA levels are frequently associated with malignant tumors, resulting in enrichment of MPE cases within the high-pCEA subgroup (19). Therefore, the increased Radscore observed in patients with high pCEA and low pADA levels does not necessarily indicate a direct biological interaction between imaging features and these biochemical markers. Rather, these biomarker thresholds may indirectly enrich populations with a higher prevalence of MPE. These findings further support the notion that the Radscore, as a high-dimensional imaging-derived feature, can maintain stable discrimination performance across different clinical subgroups by consistently identifying the underlying malignant phenotype. In this regard, the Radscore demonstrated good cross-subgroup robustness. From a biological perspective, pCEA is a glycoprotein whose concentration in PE is influenced by tumor secretory activity and cell membrane permeability (14, 20), whereas the Radscore primarily reflects spatial heterogeneity in tissue density and lesion morphology extracted from imaging data (21). The weak correlation observed between these two indicators may therefore reflect the fact that they characterize different aspects of tumor behavior.

**FIGURE 5:**
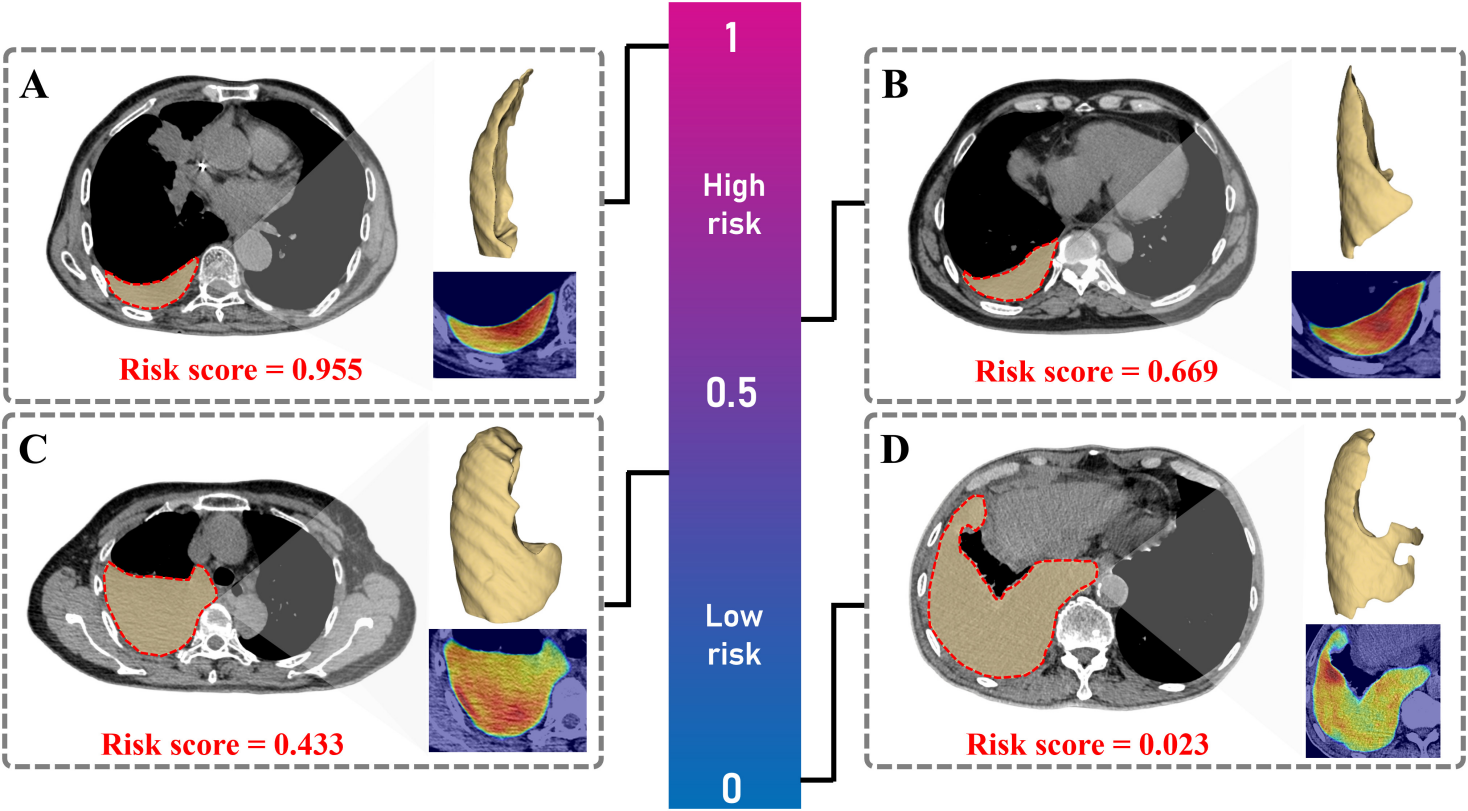
Grad-CAM and regions of interest of pleural effusion at different risk score level. **(A–D)** A CT scan with a highlighted region, a 3D anatomical rendering, and a corresponding color-coded surface map. **(A,B)** Indicate higher risk scores, 0.955 and 0.669, **(C,D)** lower scores, 0.433 and 0.023. A central vertical gradient bar demonstrates risk levels, ranging from zero (low risk, bottom) to one (high risk, top).

Among the four key features ultimately incorporated into the Radscore, three were DL-derived features and one was a wavelet-based radiomics feature (wavelet-LLH_glcm_Idmn). Although the specific anatomical or pathological interpretations of the DL features (DL_295, DL_4, and DL_80) cannot be directly visualized or intuitively explained, these features are thought to represent complex nonlinear and higher-order texture patterns captured by the neural network within high-dimensional feature space (22). This characteristic could also be partially illustrated by the Grad-CAM visualization results (Figure 5). Of greater clinical interpretability, the conventional radiomics feature wavelet-LLH_glcm_Idmn was assigned the highest coefficient in the final model (β = 0.8488). Idmn, namely the inverse difference moment normalized, is a key indicator used to quantify local image homogeneity, whereas the wavelet-LLH transformation enhances texture information at specific spatial frequency scales (23). The increased contribution of this feature suggests that MPE is characterized by reduced local gray-level uniformity and increased spatial heterogeneity on medical imaging. Therefore, this feature should not be regarded merely as a mathematical descriptor, but rather as a quantitative imaging phenotype reflecting the macroscopic pathological characteristics of MPE. The relevance of wavelet-LLH_glcm_Idmn is also consistent with the underlying rationale reported in previous radiomics studies of MPE. For example, Han et al. (9) similarly identified wavelet- and gray-level co-occurrence matrix-based features, such as wavelet_LLH_glcm_Correlation, as important predictors. The reproducibility of this category of features across different cohorts further supports the value of combining wavelet transformation with spatial texture analysis for identifying subtle malignant imaging phenotypes in MPE.

Several limitations of this study should be acknowledged. First, this was a retrospective study, and prospective investigations are still required to further confirm the diagnostic performance of the DL-radiomics model for differentiating MPE from BPE. Second, the current study was constrained by a single-center design and a relatively small validation cohort. All patients in both the training and validation sets were enrolled from the same medical center and scanned using the same equipment. Therefore, our validation cohort represents a temporal validation rather than a true external validation. Therefore, further validation using larger multicenter cohorts will be necessary before broader clinical application can be considered. Third, although no significant differences were observed in baseline characteristics, the relatively small sample size inevitably led to an imbalanced distribution of certain etiological subgroups between the training and validation sets. Notably, even though the training cohort did not include the major histological subtype, the combined model still achieved good discriminative performance (AUC = 0.864) and calibration in the validation set (calibration slope = 0.977), suggesting that the extracted Radscore and clinical biomarkers capture the pathological features of MPE rather than merely overfitting to a specific tumor histology.

## Data Availability

The original contributions presented in this study are included in the article/Supplementary material, further inquiries can be directed to the corresponding author.

## References

[1] Feller-Kopman D Light R. Pleural disease. *N Engl J Med*. (2018) 378:740–51. 10.1056/NEJMra1403503 29466146

[2] Yang Y Liu YL Shi HZ. Diagnostic accuracy of combinations of tumor markers for malignant pleural effusion: an updated meta-analysis. *Respiration*. (2017) 94:62–9. 10.1159/000468545 28427079

[3] Grosu HB Kazzaz F Vakil E Molina S Ost D. Sensitivity of initial thoracentesis for malignant pleural effusion stratified by tumor type in patients with strong evidence of metastatic disease. *Respiration*. (2018) 96:363–9. 10.1159/000490732 30016797

[4] Wang S Tian S Li Y Zhan N Guo Y Liu Y et al. Development and validation of a novel scoring system developed from a nomogram to identify malignant pleural effusion. *EBioMedicine*. (2020) 58:102924. 10.1016/j.ebiom.2020.102924 32739872 PMC7393523

[5] Wang S An J Hu X Zeng T Li P Qin J et al. A simple and efficient clinical prediction scoring system to identify malignant pleural effusion. *Ther Adv Respir Dis*. (2024) 18:17534666231223002. 10.1177/17534666231223002 38189181 PMC10775726

[6] Vukovic D Wang A Antico M Steffens M Ruvinov I van Sloun RJ et al. Automatic deep learning-based pleural effusion segmentation in lung ultrasound images. *BMC Med Inform Decis Mak*. (2023) 23:274. 10.1186/s12911-023-02362-6 38031040 PMC10685575

[7] Sexauer R Yang S Weikert T Poletti J Bremerich J Roth JA et al. Automated detection, segmentation, and classification of pleural effusion from computed tomography scans using machine learning. *Invest Radiol*. (2022) 57:552–9. 10.1097/RLI.0000000000000869 35797580 PMC9390225

[8] Cai F Cheng L Liao X Xie Y Wang W Zhang H et al. An integrated clinical and computerized tomography-based radiomic feature model to separate benign from malignant pleural effusion. *Respiration*. (2024) 103:406–16. 10.1159/000536517 38422997

[9] Han R Huang L Zhou S Shen J Li P Li M et al. Novel clinical radiomic nomogram method for differentiating malignant from non-malignant pleural effusions. *Heliyon*. (2023) 9:e18056. 10.1016/j.heliyon.2023.e18056 37539225 PMC10395353

[10] Bashour SI Mankidy BJ Lazarus DR. Update on the diagnosis and management of malignant pleural effusions. *Respir Med*. (2022) 196:106802. 10.1016/j.rmed.2022.106802 35287006

[11] Cao W Zaiane W. UCTransNet: rethinking the skip connections in U-Net from a channel-wise perspective with transformer. *Proc AAAI ACM Conf AI Ethics Soc.* (2022) 36:2441–9. 10.1609/aaai.v36i3.20144

[12] Lin J Luo J Luo Y Zhuang Y Mo T Wen S et al. Early prediction of periventricular leukomalacia from MRI changes: a machine learning approach for risk stratification. *Eur Radiol*. (2026) 36:2259–70. 10.1007/s00330-025-11981-8 40986046

[13] Cheng C Yang Y Yang W Wang D Yao C. The diagnostic value of CEA for lung cancer-related malignant pleural effusion in China: a meta-analysis. *Expert Rev Respir Med*. (2022) 16:99–108. 10.1080/17476348.2021.1941885 34112035

[14] Yang DN Niu Y Wen JX Wen XH Wang YF Yan L et al. Long-term stability of pleural fluid carcinoembryonic antigen and its effect on the diagnostic accuracy for malignant pleural effusion. *Thorac Cancer*. (2023) 14:2077–84. 10.1111/1759-7714.14996 37314828 PMC10363804

[15] Hu C Xia Y Zheng Z Cao M Zheng G Chen S et al. AI-based large-scale screening of gastric cancer from noncontrast CT imaging. *Nat Med*. (2025) 31:3011–9. 10.1038/s41591-025-03785-6 40555751 PMC12443630

[16] Wei M Zhang Y Zhao L Zhao Z. Development and validation of a radiomics nomogram for diagnosis of malignant pleural effusion. *Discov Oncol*. (2023) 14:213. 10.1007/s12672-023-00835-8 37999794 PMC10673775

[17] Xing ZC Guo HZ Hou ZL Zhang HX Zhang S. The value of computed tomography-based radiomics for predicting malignant pleural effusions. *Front Oncol*. (2024) 14:1419343. 10.3389/fonc.2024.1419343 39188676 PMC11345134

[18] Chen QY Yin SM Shao MM Yi FS Shi HZ. Machine learning-based diagnostic model for determining the etiology of pleural effusion using age, ADA and LDH. *Respir Res*. (2025) 26:170. 10.1186/s12931-025-03253-2 40316966 PMC12048966

[19] Zhang M Yan L Lippi G Hu ZD. Pleural biomarkers in diagnostics of malignant pleural effusion: a narrative review. *Transl Lung Cancer Res*. (2021) 10:1557–70. 10.21037/tlcr-20-1111 33889529 PMC8044497

[20] Ai L Wang W Li J Ye T Li Y. Use of tumor markers in distinguishing lung adenocarcinoma-associated malignant pleural effusion from tuberculous pleural effusion. *Am J Med Sci*. (2024) 368:136–42. 10.1016/j.amjms.2024.04.001 38583522

[21] Chen H Liu Y Zhao J Jia X Chai F Peng Y et al. Quantification of intratumoral heterogeneity using habitat-based MRI radiomics to identify HER2-positive, -low and -zero breast cancers: a multicenter study. *Breast Cancer Res*. (2024) 26:160. 10.1186/s13058-024-01921-7 39578913 PMC11583526

[22] Chen X Wang X Zhang K Fung KM Thai TC Moore K et al. Recent advances and clinical applications of deep learning in medical image analysis. *Med Image Anal*. (2022) 79:102444. 10.1016/j.media.2022.102444 35472844 PMC9156578

[23] Drayson OGG Montay-Gruel P Limoli CL. Radiomics approach for identifying radiation-induced normal tissue toxicity in the lung. *Sci Rep*. (2024) 14:24256. 10.1038/s41598-024-75993-y 39415029 PMC11484882

