# Supplementary material for: Incremental predictive value of a CT-based deep learning radiomics model for differentiating benign and malignant pleural effusions
Source: Front Med (Lausanne). 2026 Jul 29;13:1899111. doi: 10.3389/fmed.2026.1899111 (PMC13461531; doi:10.3389/fmed.2026.1899111)
Supplement: Supplementary file 1 [file Data_Sheet_1.docx]

Supplementary Material

# 1 Supplementary information

**S1.** **Imaging Biomarker Standardization Initiative (IBSI) reporting structure of the study**

| **Patients** | |
| --- | --- |
| Region of interest | Segmentation of the regions of interest (ROIs) was performed using 3D Slicer software (version 5.10.0; available at [www.slicer.org](http://www.slicer.org)). The location of the pleural effusion was determined on axial chest CT images. |
| Patient Preparation | No special patient preparation (e.g., fasting) was required. All patients underwent a standard chest CT examination in accordance with institutional protocols. |
| Computed tomography (CT) developing agent | No intravenous or oral contrast agents were administered |
| **Acquisition and Reconstruction** | |
| Protocol | CT examination: All patients underwent CT scanning using a 64-slice CT scanner (LightSpeed VCT, GE Healthcare), with the scan range extending from the thoracic inlet to the inferior margin of the bilateral adrenal glands. Scanning parameters were as follows: tube voltage, 120 kV; automatic tube current modulation; pitch, 0.8; collimation, 128 × 0.625 mm; slice thickness, 5 mm; and slice interval, 5 mm. Thin-section images were reconstructed from the raw data with a slice thickness and interval of 1.25 mm. |
| Scanner type | Image data were acquired using a 64-slice computed tomography scanner (LightSpeed VCT, GE). |
| **Delineation** | |
| Software | 3D Slicer (version 5.10.0; www.slicer.org) |
| ROI definition | In 3D Slicer, regions of interest (ROIs) were defined using manual segmentation. The “Paint” and “Erase” tools were applied slice-by-slice for fine adjustments to ensure accuracy. |
| Number of experts | To assess inter-observer reproducibility, all images were independently segmented by two radiologists: one junior radiologist (6 years of experience) and one senior radiologist (13 years of experience). |
| Reference image | CT |
| **Radiomics feature extraction** | |
| Software | Python (version 3.9) |
| Package | PyRadiomics (https://github.com/AIM-Harvard/pyradiomics); PyTorch (https://pytorch.org/get-started/locally) |
| Method | Images were resampled to 1 mm³ isotropic voxels using B-spline interpolation, with a bin width of 5. ROI masks were resampled using nearest-neighbor interpolation. Considering that pleural effusion and pleural lesion regions may contain tissues with mixed densities, an absolute threshold resegmentation strategy was applied, with CT attenuation values restricted to a range of −20 to 80 HU. Because Hounsfield units (HU) on CT images reflect the absolute physical density of tissue attenuation, global gray-level normalization was disabled to preserve the original physical meaning and absolute zero point of the images (voxel array shift = 0). Radiomics features were extracted using PyRadiomics, including shape features, first-order statistics, gray-level co-occurrence matrix (GLCM), gray-level run-length matrix (GLRLM), gray-level size zone matrix (GLSZM), gray-level dependence matrix (GLDM), and neighboring gray-tone difference matrix (NGTDM). Feature extraction was performed on original images, wavelet-transformed images, and Laplacian of Gaussian (LoG)-filtered images. |
| Discretization | Uniform (equal-width binning) |
| Bin width | 5 for CT |
| Kernels of the filter | Wavelet: coif1 wavelet basis; LoG: sigma values of 1.0, 2.0, 3.0, 4.0, and 5.0 mm; Square, Square Root, Logarithm, Exponential: pixel-wise intensity transformations (no spatial kernel) |
| Biomarker set | First Order Statistics, Shape (3D), Gray Level Co-occurrence Matrix (GLCM), Gray Level Run Length Matrix (GLRLM), Gray Level Size Zone Matrix (GLSZM), Neighboring Gray Tone Difference Matrix (NGTDM), Gray Level Dependence Matrix (GLDM) |
| Exclusion criteria | ICC smaller than 0.75 |

S2. TRIPOD+AI (Transparent Reporting of a multivariable prediction model for Individual Prognosis Or Diagnosis + Artificial Intelligence

S3. Deep Learning Feature Extraction

Each downsampling and upsampling block consisted of two grouped convolution layers. Every grouped convolution included a 3 × 3 convolution kernel, followed sequentially by batch normalization and a ReLU activation layer. Network parameters were optimized using the Adam optimizer with an initial learning rate of 0.0001 and a batch size of 32. To provide supervision for the network, manual 3D annotations delineated by a radiologist with 6 years of clinical experience were utilized as ground-truth masks. The combined binary cross-entropy loss and Dice loss between the network predictions and these manual masks were employed as the loss function to train the network. The DL feature extraction procedure was strictly designed to prevent data leakage and bridge the dimensionality from 3D ROIs to patient-level features. The procedure was as follows:

i) 3D to 2D slice extraction and per-slice normalization: Given that the DL network operates on 2D inputs, the 3D rectangular ROIs of each patient were sliced along the z-axis. All 2D slices containing the pleural effusion lesion were extracted, scaled to a unified size of 224 × 224 pixels, and imported as input images. Maximum-minimum normalization was carried out independently on a per-slice basis according to the formula shown in Equation [1], where X represents the grayscale value in the original image, X_M_ represents the maximum grayscale value in the original image, X_m_ represents the minimum grayscale value in the original image and X* represents the grayscale value in the normalized image. This slice-independent normalization completely avoided cohort-level pixel information leakage.

$$X^{*}=\left( \frac{X -X_{m}}{X_{M}-X_{m}} \right)[1]$$

ii) Slice-level feature extraction: The normalized 2D images were input to the trained DL network. Feature maps were extracted from the fourth downsampling activation layer of UCTransNet. Global average pooling was performed to acquire a semantic segmentation feature vector with a 1 × 512 dimension for each individual 2D slice.

iii) Patient-level dimensionality reduction via Bimodal Clustering: Since pleural effusion lesions exhibit spatial heterogeneity, where core slices contain stable lesion signals, while marginal/apical slices often suffer from artifacts or partial volume effects, we hypothesized a bimodal distribution of slice features. For each patient, an unsupervised K-Means clustering algorithm (K = 2) was applied to their complete set of slice-level features, separating them into a "core signal" cluster and a "marginal noise" cluster.

iv) Dynamic reference matching and anomaly rejection: To select the most representative feature cluster while strictly preventing data leakage, a "Global Reference Vector" representing the typical lesion phenotype was established. The L_2_ Euclidean distance between the two aforementioned clusters and this reference vector was then evaluated. The cluster closest to the reference vector was retained, and its mean vector was utilized as the final definitive 1 × 512 DL feature for that patient.

**Supplementary Tables**

**Table S1.** **Baseline characteristics of BPE and MPE**

| **Variables** | **Total**  **(n = 260)** | **BPE**  **(n = 159)** | **MPE**  **(n = 101)** | ***P*** |
| --- | --- | --- | --- | --- |
|  |  |  |  |  |
| **Age (years)** | 71.26 ± 10.46 | 71.35 ± 11.63 | 71.12 ± 8.33 | 0.851 |
| **Gender (%)** |  |  |  | 0.637 |
| Female | 106 (40.77) | 63 (39.62) | 43 (42.57) |  |
| Male | 154 (59.23) | 96 (60.38) | 58 (57.43) |  |
| **Smoking (%)** |  |  |  | 0.810 |
| No | 217 (83.46) | 132 (83.02) | 85 (84.16) |  |
| Yes | 43 (16.54) | 27 (16.98) | 16 (15.84) |  |
| **Etiology (%)** |  |  |  | <0.001 |
| Poorly differentiated | 1 (0.38) | 0 (0.00) | 1 (0.99) |  |
| Squamous carcinoma | 3 (1.15) | 0 (0.00) | 3 (2.97) |  |
| Adenocarcinoma | 89 (34.23) | 0 (0.00) | 89 (88.12) |  |
| Small cell carcinoma | 2 (0.77) | 0 (0.00) | 2 (1.98) |  |
| Breast cancer | 5 (1.92) | 0 (0.00) | 5 (4.95) |  |
| Lymphoma | 1 (0.38) | 0 (0.00) | 1 (0.99) |  |
| Tuberculosis | 41 (15.77) | 41 (25.79) | 0 (0.00) |  |
| Parapneumoniceffusion | 76 (29.23) | 76 (47.80) | 0 (0.00) |  |
| Heart failure | 11 (4.23) | 11 (6.92) | 0 (0.00) |  |
| Cirrhosis | 22 (8.46) | 22 (13.84) | 0 (0.00) |  |
| Other benign diseases | 9 (3.46) | 9 (5.66) | 0 (0.00) |  |
| **Leukocyte (10^9^/L)** | 6.29 (5.15, 7.79) | 6.29 (5.15, 7.79) | 6.29 (5.15, 7.79) | 0.785 |
| **Neutrophil (10^9^/L)** | 5.05 (3.90, 7.30) | 5.05 (3.90, 7.30) | 5.05 (3.90, 7.30) | 0.132 |
| **Monocyte (10^9^/L)** | 0.48 (0.34, 0.63) | 0.48 (0.34, 0.63) | 0.48 (0.34, 0.63) | 0.228 |
| **Lymphocyte (10^9^/L)** | 0.90 (0.60, 1.30) | 0.90 (0.60, 1.30) | 0.90 (0.60, 1.30) | 0.747 |
| **Platelet (10^9^/L)** | 178.00 (134.00, 248.00) | 178.00 (134.00, 248.00) | 178.00 (134.00, 248.00) | 0.010 |
| **Albumin (g/L)** |  |  |  | 0.481 |
| ≤ 55 | 254 (97.69) | 154 (96.86) | 100 (99.01) |  |
| > 55 | 6 (2.31) | 5 (3.14) | 1 (0.99) |  |
| **sLDH (U/L)** |  |  |  | 0.092 |
| ≤ 250 | 178 (68.46) | 115 (72.33) | 63 (62.38) |  |
| > 250 | 82 (31.54) | 44 (27.67) | 38 (37.62) |  |
| **sCEA (ng/mL)** |  |  |  | <.001 |
| ≤ 5 | 182 (70.00) | 131 (82.39) | 51 (50.50) |  |
| > 5 | 78 (30.00) | 28 (17.61) | 50 (49.50) |  |
| **sCA125 (U/mL)** |  |  |  | <.001 |
| ≤ 35 | 160 (61.54) | 119 (74.84) | 41 (40.59) |  |
| > 35 | 100 (38.46) | 40 (25.16) | 60 (59.41) |  |
| **sCA199 (ng/mL)** |  |  |  | <.001 |
| ≤ 37 | 226 (86.92) | 147 (92.45) | 79 (78.22) |  |
| > 37 | 34 (13.08) | 12 (7.55) | 22 (21.78) |  |
| **pCEA (ng/mL)** |  |  |  | <.001 |
| ≤ 5 | 180 (69.23) | 139 (87.42) | 41 (40.59) |  |
| > 5 | 80 (30.77) | 20 (12.58) | 60 (59.41) |  |
| **pLDH (U/L)** |  |  |  | 0.059 |
| ≤ 274 | 93 (35.77) | 64 (40.25) | 29 (28.71) |  |
| > 274 | 167 (64.23) | 95 (59.75) | 72 (71.29) |  |
| **pADA (U/L)** |  |  |  | <.001 |
| ≤ 25.74 | 173 (66.54) | 82 (51.57) | 91 (90.10) |  |
| > 25.74 | 87 (33.46) | 77 (48.43) | 10 (9.90) |  |
| **Radscore** | -0.45 (-1.52, 0.43) | -0.45 (-1.52, 0.43) | -0.45 (-1.52, 0.43) | <.001 |

**Table S2. Reclassification and Discrimination Improvement of the Combined Model (Clinical Model + Radscore) Compared with the Clinical Model Alone**

| **Metrics** | **Estimate** | **SE** | **Z** | **95% CI** | ***P*** |
| --- | --- | --- | --- | --- | --- |
| **Continuous NRI** |  |  |  |  |  |
| Overall NRI | 0.588 | 0.262 | 2.241 | 0.074, 1.102 | 0.025 |
| NRI for events | 0.455 | 0.190 | 2.394 | 0.082, 0.827 | 0.017 |
| Increase for events | 72.7% | - | - | - | - |
| Decrease for events | 27.3% | - | - | - | - |
| NRI for non-events | 0.133 | 0.181 | 0.737 | -0.221, 0.488 | 0.461 |
| Decrease for non-events | 43.3% | - | - | - | - |
| Increase for non-events | 56.7% | - | - | - | - |
| **IDI** |  |  |  |  |  |
| Overall IDI | 0.183 | 0.053 | 3.430 | 0.079, 0.288 | <0.001 |
| Mean change in events | 0.106 | - | - | - | - |
| Mean change in non-events | 0.078 | - | - | - | - |

**Table S3. Diagnostic performance of the Clinical Model and the Combined Model (Clinical Model + Radscore) in the training, testing, external validation, and internal validation cohorts.**

| **Dataset** | **AUC (95% CI)** | **Accuracy** | **Sensitivity**  **(95%CI)** | **Specificity**  **(95%CI)** |
| --- | --- | --- | --- | --- |
| **Clinical Model** |  |  |  |  |
| **Training set** | 0.873  (0.815, 0.930) | 0.808 | 0.839  (0.717, 0.924) | 0.789  (0.690, 0.868) |
| **Testing set** | 0.860  (0.769, 0.950) | 0.774 | 0.826  (0.612, 0.950) | 0.744  (0.579, 0.870) |
| **Validation set** | 0.875  (0.783, 0.968) | 0.808 | 0.909  (0.708, 0.989) | 0.733  (0.541, 0.877) |
| **Combined Model** |  |  |  |  |
| **Training set** | 0.922  (0.880，0.965) | 0.829 | 0.875  (0.759, 0.948) | 0.800  (0.702, 0.877) |
| **Testing set** | 0.929  (0.867，0.990) | 0.871 | 0.870  (0.664, 0.972) | 0.872  (0.726, 0.957) |
| **Validation set** | 0.944  (0.877，1.000) | 0.904 | 0.955  (0.772, 0.999) | 0.867  (0.693, 0.962) |

**Table S4. Pairwise Comparison of Model Discrimination Across Cohorts**

| **Model** | **AUC**  **(95% CI)** | ***P*_Delong_** |
| --- | --- | --- |
| **Training** |  |  |
| **Clinical model** | 0.873  (0.815, 0.930) | - |
| **Radscore** | 0.805  (0.733, 0.877) | 0.136 |
| **Combined model** | 0.922  (0.880，0.965) | 0.005 |
| **Testing** |  |  |
| **Clinical model** | 0.860  (0.769, 0.950) | - |
| **Radscore** | 0.805  (0.696, 0.914) | 0.436 |
| **Combined model** | 0.929  (0.867，0.990) | 0.025 |
| **Validation** |  |  |
| **Clinical model** | 0.875  (0.783, 0.968) | - |
| **Radscore** | 0.864  (0.766, 0.962) | 0.862 |
| **Combined model** | 0.944  (0.877，1.000) | 0.053 |

**Table S5. Quantitative Calibration Metrics of Different Models**

| **Model** | **Calibration Intercept** | **Calibration Slope** | **Brier Score** |
| --- | --- | --- | --- |
| **Training** |  |  |  |
| **Radscore** | <0.001 | 1.000 | 0.173 |
| **Clinical** | <0.001 | 1.000 | 0.137 |
| **Combined** | <0.001 | 1.000 | 0.110 |
| **Testing** |  |  |  |
| **Radscore** | -0.264 | 1.014 | 0.176 |
| **Clinical** | -0.288 | 0.922 | 0.153 |
| **Combined** | -0.357 | 0.913 | 0.121 |
| **Validation** |  |  |  |
| **Radscore** | 0.398 | 1.586 | 0.157 |
| **Clinical** | -0.262 | 0.901 | 0.157 |
| **Combined** | -0.209 | 0.977 | 0.112 |

**Supplementary figures**

**Figure S1. Top 20 feature stability rankings from 10-fold nested cross-validation (A); unbiased AUC distribution from 10-fold nested cross-validation (B)**

**Figure S2. Flowchart of case inclusion and exclusion.**

**Figure S3. Schematic illustration of region-of-interest (ROI) delineation. (A) Malignant pleural effusion with atelectasis; (B) ROI delineated by a junior physician; (C) ROI delineated by a senior physician; (D) benign pleural effusion secondary to heart failure with concomitant pneumonia; (E) ROI delineated by a junior physician; (F) ROI delineated by a senior physician.**

**Figure S4. Receiver operating characteristic (ROC) curves of the Radscore and blood/pleural effusion biochemical biomarkers. Model 1: Radscore; Model 2: serum lactate dehydrogenase; Model 3: serum carcinoembryonic antigen; Model 4: serum cancer antigen 125; Model 5: serum cancer antigen 19-9; Model 6: pleural effusion carcinoembryonic antigen; Model 7: pleural effusion lactate dehydrogenase.**
